# Abnormal expression of *ATP1A1* and *ATP1A2* in breast cancer

**DOI:** 10.12688/f1000research.10481.1

**Published:** 2017-01-05

**Authors:** Alexey Bogdanov, Fedor Moiseenko, Michael Dubina

**Affiliations:** 1 St Petersburg Academic University, St. Petersburg, Russian Federation; 2 Practical Center for Specialized Types of Medical Care (Oncologic), St-Petersburg, Russian Federation; 3 Peter the Great St. Petersburg Polytechnic University, St. Petersburg, Russian Federation; 4 The Petersburg Nuclear Physics Institute, Gatchina, Russian Federation

**Keywords:** breast cancer, Na+/K+-ATPase, gene expression, abnormality, ATP1A1, ATP1A2

## Abstract

Breast cancer is the first in incidence and the second in death among all solid tumors occurring in women. The identification of molecular genetic abnormalities in breast cancer is important to improve the results of treatment. In the present study, we analyzed microarray data of breast cancer expression profiling (NCBI GEO database, accession GSE65194), focusing on Na ^+^/K ^+^-ATPase coding genes. We found overexpression of the *ATP1A1* and down-regulation of the *ATP1A2*. We expect that our research could help to improve the understanding of predictive and prognostic features of breast cancer.

## Introduction

Breast cancer is one of the most common and deadly female solid tumors ^1^. According to reports from Perou *et al*. ^2^, further confirmed by other investigators ^3, 4^, breast cancer is a highly molecularly heterogeneous disease. The identification of molecular genetic abnormalities in breast cancer is important to improve the results of treatment and, for instance, to reveal new targets for specific therapies. Recent studies based on original retrospective analysis of digitalis use in breast cancer patients have demonstrated the anticancer effect of cardiac glycosides ^5^ that directly inhibit Na ^+^/K ^+^-ATPase (NKA) activity. NKA signaling functions after interaction with cardiac glycosides were also shown ^6^. It seems rational that expression of NKA might influence breast cancer prognosis.

NKA is a significant integral membrane protein. NKA’s main function is the creation and maintenance of electrochemical gradients for sodium and potassium ions in the living cell. These gradients have critical importance for control of cell volume, osmolarity and resting potential ^7, 8^. The minimal functional NKA consists of two associated alpha- and beta- subunits. The catalytic alpha-subunit is responsible for conversion of ATP energy to transport of Na ^+^ and K ^+^ across cell membranes and has ATP and cardiac glycosides binding sites. It may be present in human tissues in four different isoforms (α1, α2, α3, α4 – found only in testicles). The beta-subunit is responsible for delivery and insertion of alpha one in cell membranes and has three distinct isoforms in humans (β1, β2, β3) ^8– 10^. NKA subunits are variably expressed in different human tissues ^11^. Changes in the relative expression between different isoforms are associated with a number of pathological processes including malignant transformation ^12, 13^. Both down- and up-regulation of alpha- and beta- subunits were shown in solid tumors of different origin ^14– 19^.

In the present study, we analyzed public breast cancer expression profiles made using Affymetrix Human Genome U133 Plus 2.0 Array (NCBI GEO database ^20^, accession GSE65194) for the expression of alpha subunits of NKA. We found abnormalities in *ATP1A1* (coding α1-subunit) and *ATP1A2* (coding α2-subunit) expression ( Table 1) in breast cancer samples relative to their expression in normal breast tissue. *ATP1A1* was overexpressed approximately 1.5 times in all groups of breast cancer samples (p<0.05). Coincidently, *ATP1A2* expression decreased by more than 2 times (p<0.05). There were no differences observed in the expression of *ATP1A3* (coding α3-subunit).

**Table 1.** NKA genes expression in breast cancer samples relative to normal breast tissue.

Breast cancer group	Lum A	Lum B	Her2	TNBC
Gene	Relative expression/(ANOVA P-value)			
*ATP1A1*	1.53 (0.009016)	1.38 (0.04454)	1.66 (0.005926)	1.44 (0.015725)
*ATP1A2*	-2.49 (1.85·10 ^-07^)	-2.52 (8.50·10 ^-09^)	-2.78 (5.48·10 ^-08^)	-2.87 (2.08·10-11)
*ATP1A3*	-1.05 (0.429089)	1.03 (0.308298)	-1.04 (0.768041)	-1.04 (0.527878)

## Methods

Preanalytical procedures consisted of a robust multichip analysis (RMA) algorithm ^21^, including background correction, probe set signal integration, and quantile normalization. For this purpose, we used Expression Console 1.4 software (Affymetrix, Inc. USA). We utilized Transcriptome Analysis Console 3.0 software (Affymetrix, Inc. USA) to analyze the obtained CHP files and to detect differentially expressed genes using one-way between subjects ANOVA. Array data for 41 triple negative samples (TNBC group), 30 Her2-positive (Her2 group), 30 Luminal B (Lum B group), 29 Luminal A (Lum A group) breast cancer samples and 11 normal breast tissue samples were investigated.

## Conclusions

Using a public microarray dataset we found abnormalities in the expression of *ATP1A1* and *ATP1A2* in breast cancer samples. This may correlate with digitalis anticancer activity, but requires additional research. We expect that our research could help to improve the understanding of predictive and prognostic features of breast cancer.

## Data and software availability

Raw data for Table 1 are available at:

https://www.ncbi.nlm.nih.gov/geo/download/?acc=GSE65194&format=file ^22^.

Expression Console 1.4 software and Transcriptome Analysis Console 3.0 software (Affymetrix, Inc. USA) are available after free customer registration at:

http://www.affymetrix.com/support/technical/software_downloads.affx.
