# Supplementary material for: Abnormal expression of ATP1A1 and ATP1A2 in breast cancer
Source: F1000Res. 2017 Jan 5;6:10. [Version 1] doi: 10.12688/f1000research.10481.1 (PMC5428514; doi:10.12688/f1000research.10481.1)
Supplement: Peer review history

# Peer review: Referee response for version 1

**DOI:** 10.5256/f1000research.11294.r21728

**Published:** 2017-05-10

**Authors:** Jen-Tsan Ashley Chi

**Affiliations:** 1 Department of Molecular Genetics and Microbiology, Duke University Medical Center, Durham, NC, 27708, USA

I think the analysis is appropriate to examine the relative expression of the *ATP1A1 *and *ATP1A2* among different breast cancer cells. The data analysis is standard and appropriate. One helpful thing is to validate the findings in other breast cancer expression datasets beyond this discovery dataset. Another relevant thing is whether the abnormal expression of these genes are associated with varying clinical outcomes.

I have read this submission. I believe that I have an appropriate level of expertise to confirm that it is of an acceptable scientific standard.

# Peer review: Referee response for version 1

**DOI:** 10.5256/f1000research.11294.r20402

**Published:** 2017-02-21

**Authors:** Mikhail Fedyanin

**Affiliations:** 1 Department of Clinical Pharmacology and Chemotherapy, N.N. Blokhin Russian Cancer Research Center, Moscow, Russian Federation

Over the past years several papers were published concerning prognostic role of *ATP1A1* expression in hepatocellular carcinoma, lung cancer, and esophageal cancer. The authors of the present study show that increased expression of *ATP1A1* observed at all breast cancer phenotypes compared to normal tissue.

 I would like to note that the authors studied gene expression only, but did not appreciate the immunohistochemical (IHC) changes in the content of gene products. In the absence of data of the IHC expression of *ATP1A1*, it is desirable to represent the differences in gene expression of *ATP1A1* compared to referent genes for membrane transporters ( http://bmcmolbiol.biomedcentral.com/articles/10.1186/1471-2199-7-29 ). Given a sufficiently large number of patients included in the study, it is interesting to evaluate the prognostic and predictive value of these findings. But I can conclude that this article is interesting for medical oncologists and molecular biologists.

I have read this submission. I believe that I have an appropriate level of expertise to confirm that it is of an acceptable scientific standard.
